# Lower limb pain among workers: a cross-sectional analysis of the fifth European Working Conditions Survey

**DOI:** 10.1007/s00420-017-1220-4

**Published:** 2017-04-17

**Authors:** Maria-Gabriela Garcia, Margaret Graf, Thomas Läubli

**Affiliations:** 1 Sensory-Motor Systems Lab, Department of Health Sciences and Technology, ETH Zürich, Sonneggstrasse 3, ML G53.2, 8092 Zurich, Switzerland; 2 State Secretariat of Economic Affairs SECO, Bern, Switzerland; 3 Institute of Occupational and Social Medicine and Health Services Research, University of Tübingen, Tubingen, Germany

**Keywords:** Work-related, Musculoskeletal disorders, Risk indicator, Exposure, Prevalence, Lower limb

## Abstract

**Objective:**

Develop a model to predict the prevalence of lower limb pain using indicators of high workplace exposures based on the fifth European Working Conditions Survey, evaluate its impact and explore its significance for work-related health problems.

**Method:**

Cross-sectional interview data of 35,372 workers from 27 countries of the European Union in 2010 (EU27) were used to develop (20% sample) and validate (80% sample) a logistic regression model for lower limb pain. Independent variables included descriptions of working conditions, assessments of physical and psychosocial exposures at work, and demographic factors. The impact of the model was explored through the amount of lower limb pain cases attributable to work and estimating work absences correlated with lower limb pain.

**Results:**

The resulting logistic model included ten risks indicators and one preventive factor. The highest odds ratios (OR) corresponded to “tiring or painful positions” OR 2.0, 99% confidence interval (99% CI) 1.9–2.2, and “not satisfied with level of working conditions in the job” (OR 1.6, 99% CI 1.5–1.7). The prevalence of work-related lower limb pain was 16.5% for men and 15.8% for women for the 27 countries of the European Union. Estimates based on the developed model revealed more than 34 million cases of work-related lower limb pain, where four physical risks explained about 22 million cases. In addition, more than 3 million days of absence from work in 2010 could be attributed to lower limb pain.

**Conclusion:**

Lower limb pain is highly prevalent among the European workforce and work exposures are a major contributing factor. Effective workplace interventions should aim at improving working conditions at workplaces with multiple risks.

## Introduction

In Europe, work-related musculoskeletal disorders represent a substantial economic burden to society and are one of the major causes of health-related productivity loss (Bevan 2015). Besides having obvious consequences for the individual, work-related health problems have substantial costs for their employers (Oh et al. 2011). Many studies have investigated the prevalence of and risk factors for work-related disorders of the upper limbs, neck and low back. However, work-related lower limb symptoms have received less attention when compared to other work-related musculoskeletal symptoms in the upper body or low back pain.

A small number of studies present evidence that working populations such as nurses, assembly line workers, industrial workers and service and sales workers report work-related lower limb symptoms (Andersen et al. 2007; Chee and Rampal 2004; Montano 2014; Roelen et al. 2008; Stolt et al. 2016). The prevalence of lower limb pain in these studies varied from 16 to 50%. Several physical risk factors such as heavy pushing or pulling, standing and regular lifting have been associated with lower limb pain as well as psychosocial factors (Andersen et al. 2007; Dawson et al. 2002; Roelen et al. 2008; Yue et al. 2014). In general, most epidemiology studies of the lower limbs focus on the analysis of risk indicators in specific work tasks. However, to our knowledge, a broader evaluation of working conditions associated with lower limb pain has not been performed. Thus, clear risk indicators are missing to evaluate the impact of lower limb symptoms in a large population.

The purpose of this study is to develop a model based on the very large fifth European Working Conditions Survey (EWCS) to predict the prevalence of lower limb pain as a function of indicators of high exposures at work. The research questions were:

- Which are the most important working-conditions indicators correlated with a high prevalence of lower limb pain?
- How many cases including lower limb pain can be attributed to exposures at work in the EU?
- What is the association of work-related lower limb pain with other health problems, absenteeism and expectations regarding ability to continue to do the same job in the future?

## Method

### Population and setting

The present study analyzed data from the fifth EWCS carried out in 2010. The survey was undertaken in 27 European Union Member States (EU) and seven further European countries not included in this study. The target population was EU residents aged 15 and over who worked for payment for at least 1 h in the week prior to the survey. A multi-stage random stratified sample was performed to identify participants. A random sample of households was drawn from a random section of each country that was divided according to degree of urbanization. In each household, the person with current employment and with the most recent birthday was interviewed face to face, for about 44 min and using a questionnaire in the national language of the country. The survey consisted of 325 questions on the quality of employment, working conditions and health status of the interviewee. In total 35,372 interviews were completed. The overall response rate was 44% (Parent-Thirion et al. 2012). A detailed description of the standardized questionnaire and the methods has been published by Eurofound (2010).

### Working-condition variables

The survey covered multiple aspects of work such as physical factors, psychosocial factors, work organization, financial security, among others, classified into different topics by Eurofound (2010). In addition, questions regarding household characteristics and demographics were included. A detailed list of the classification of the variables into topics has been published elsewhere (Eurofound 2010). This study uses the working-condition variables and that topic classification. These variables are not considered to be risk factors that directly predict a higher prevalence of symptoms; however, this study assumes that they identify work situations associated with health risks.

### Case definition

Several EWCS questions are related to health problems over the last 12 months before the interview (health outcome variables). In this study the variable of interest was the presence of muscular pains in the lower limbs, hereafter referred to as lower limb pain. This variable corresponds to the survey question: “Over the last 12 months, did you suffer from muscular pain in lower limbs (hips, legs, knees, feet, etc.)?” The answers were: yes/no.

### Analysis

The analytical procedure of this study has three steps: (1) development of a logistic regression model; (2) validation of the model; (3) exploring the impact of the derived model. In summary, 20% of the interviews were randomly selected and stratified by country to form a subsample data set of 7083 interviews. The subsample was used to develop a statistical model that was subsequently validated using the rest of the data. From the subsample, questions with missing responses of over 5% were excluded. In addition, questions concerning information about the respondent household members and those not related to working conditions were not included. The variables related to health outcomes were used only in the third step of the analytical procedure; impact evaluation. The responses for each of the remaining 161 questions were dichotomized with 1 representing high exposure and 0 none, low or moderate exposure. Dichotomization for all questions that involve Likert scales was done through grouping the side of the scale with 20–25% of the responses representing the negative aspect or high exposures into the “exposed group” (1) and all other responses into the “controls” (0). Variables that presented a skewed distribution with less than 10% of responses on either side were discarded.

A Chi-square goodness of fit test was performed to test for correlations of the 133 remaining variables with the lower limb pain outcome variable. Sixty-four variables with a phi coefficient equal or lower than 0.05 were discarded from further analysis. The remaining variables were grouped according to the topics specified by Eurofound, with the exception of the variables related to health outcomes. Thus, 46 variables grouped into 21 topics were used for a bivariate analysis and per group multivariate modelling in relation to lower limb pain. Odds ratios (OR) and confidence limits (CL) were estimated using logistic regression techniques with backward elimination for each group at *p* = 0.05. The variables that were not eliminated in the group logistic regression procedure were entered into a new model and tested again using logistic regression with backward elimination to determine the final model at *p* = 0.01. This model was correlated with three demographic variables; age, gender and level of education. Age was classified into two categories, <50 and ≥50 years of age, with higher age considered as “exposed group”. For the level of education, high exposure was defined as having no or only primary education (ISCED 1). For gender, females (1) were compared with males (0).

For the second step of the analytical procedure, the developed model was tested on the rest of the data set (80%). The sample size for this step was 28,289. The OR and CL of both subsamples were compared to check the stability of the model.

For the third and last step, exploring the impact of the derived model, the complete EWCS 2010 data set (*n* = 35,372) was used. By means of frequency analysis, the number of lower limb pain cases attributable to work was calculated for each gender depending on the number of risks indicators present in each case. For this analysis, the variables of the model with positive associations with lower limb pain were used and the single subjects were weighted according to the cross-national selection probability weighting performed by Eurofound (2010). The frequency analysis considered the presence of risk indicators from 1 to 5 and ≥6: Only a few respondents were exposed to more than six predictors included in the model. The frequency of lower limb pain cases without any risk indicator was used as the reference for calculating those cases attributable to work. In addition, a similar procedure was performed for those risk indicators related to physical load; defined as the involvement of mechanical forces generated by the human body. An estimate of the work-related lower limb pain cases and those cases due to physical load were calculated using the employment statistics of the 27 countries of the EU in 2010 (Eurostat 2010). Furthermore, relative risks were calculated by the ratio of the prevalence of an event when exposed to the prevalence of the event when not exposed.

The complete EWCS 2010 dataset was used to explore the association of lower limb pain with other health outcome variables through bivariate correlations and ascertain the number of cases with both symptoms. Factors like country of residence, socioeconomic characteristics, occupational groups, etc., were not considered; however, the analysis was adjusted by age and gender. The expectation of the respondents toward being able to do the same job when reaching 60 years of age was compared between those that reported lower limb pain and those who did not. Furthermore, absenteeism from work due to health problems and due to an accident at work was investigated among respondents with and without lower limb pain. The amount of days of absence from work over the year was estimated by the difference between the mean of absenteeism of respondents with lower limb pain and those without.

All statistical analyses were performed with SAS 9.3 (SAS Institute, North Carolina, USA). The variable names present in this study were shortened for presentation purposes. The complete variable question and its number according to the EWCS 2010 survey can be found at Eurofound site.

## Results

### Model development

#### Bivariate results

Table 1 presents the bivariate analysis of lower limb pain with the 46 variables and 21 topics. The topics were sorted in descending order according to the variable with the highest OR. Within each topic, data is also presented from the highest to lowest OR. Thirteen topics included only one variable. The topics with the highest OR for lower limb pain were ergonomic issues, work satisfaction and job hazards. The three highest OR among all variables were: tiring and painful positions, carrying or moving heavy loads and dissatisfaction with working conditions.

**Table 1:** Correlations between lower limb pain and working conditions from the fifth European Working Conditions Survey 2010 on 27 EU countries

Variable	Bivariate analysis			Analysis per topic			Topics
	OR	95% LCL	95% UCL	OR	95% LCL	95% UCL	
Tiring or painful positions	3.03	2.71	3.39	2.17	1.91	2.46	Ergonomic issues
Carrying or moving heavy loads	2.74	2.43	3.10	1.69	1.48	1.94	Ergonomic issues
Standing	2.19	1.96	2.44	1.42	1.26	1.61	Ergonomic issues
Repetitive hand or arm movements	1.79	1.58	2.01				Ergonomic issues
Lifting or moving people	1.34	1.17	1.53	1.17	1.01	1.34	Ergonomic issues
Working with computers	0.50	0.44	0.58	0.79	0.65	0.95	Ergonomic issues
Using internet/email for professional purposes	0.49	0.43	0.55	0.69	0.58	0.83	Ergonomic issues
Dissatisfaction with working conditions	2.45	2.16	2.78	2.45	2.16	2.78	Work satisfaction
High temperatures	2.43	2.17	2.73	1.63	1.42	1.87	Job hazards
Breathing in smoke, fumes, powder or dust	2.28	2.01	2.58	1.19	1.01	1.40	Job hazards
Low temperatures	2.17	1.94	2.43	1.34	1.17	1.54	Job hazards
Vibrations from hand tools, machinery, etc.	2.15	1.92	2.41	1.22	1.05	1.41	Job hazards
Loud noise	2.13	1.89	2.41	1.25	1.08	1.45	Job hazards
Breathing in vapors	1.95	1.74	2.18	1.21	1.05	1.40	Job hazards
Contact with chemical products	1.92	1.68	2.19	1.26	1.08	1.47	Job hazards
Tobacco smoke from others	1.56	1.39	1.75				Job hazards
Contact with materials which can be infectious	1.55	1.38	1.75				Job hazards
Disagree: “I am well paid for the work I do”	1.98	1.78	2.20	1.66	1.47	1.88	Job security
Disagree: “My job offers good prospects for career advancement”	1.91	1.68	2.17	1.47	1.26	1.71	Job security
Disagree: I feel ‘at home’ in this organisation	1.63	1.40	1.89				Job security
Disagree: it would be easy for me to find a job of similar salary	1.57	1.39	1.76	1.42	1.24	1.62	Job security
Disagree: the organisation I work for motivates me to give my best job performance	1.55	1.36	1.78	1.20	1.03	1.40	Job security
Agree: I might lose my job in the next 6 months	1.48	1.30	1.68	1.26	1.10	1.45	Job security
Required to wear personal protective equipment	1.74	1.58	1.93	1.74	1.58	1.93	Protective equipment^a^
Working at very high speed	1.72	1.53	1.93	1.74	1.55	1.95	Pace of work
Working to tight deadlines	1.31	1.17	1.47				Pace of work
Working hours do not fit well with family or social commitments	1.71	1.51	1.94	1.62	1.43	1.85	Work-life balance
Very difficult to take an hour or two off during working hours	1.49	1.30	1.72	1.37	1.19	1.58	Work-life balance
Subjected to verbal abuse at work	1.57	1.35	1.83	1.57	1.35	1.83	Verbal abuse^a^
Mostly experience stress in the work	1.54	1.38	1.72	1.54	1.38	1.72	Work pressure^a^
Work 6 or 7 days per week in main job	1.54	1.36	1.74	1.54	1.36	1.74	Working days per week^a^
Short repetitive tasks of less than 10 min	1.52	1.37	1.68	1.52	1.37	1.68	Repetitive tasks
Short repetitive tasks of less than 1 min	1.32	1.18	1.47				Repetitive tasks
Main place of work is not employers’ or own business’ premises	1.51	1.35	1.69	1.51	1.35	1.69	Place of work^a^
Work on Saturdays more than 2 times a month	1.47	1.31	1.65	1.40	1.24	1.58	Working time
Work in the evening more than five times a month	1.30	1.16	1.47	1.19	1.06	1.35	Working time
Work on Sundays more than one time a month	1.29	1.15	1.45				Working time
Own mistakes could cause physical injury to others	1.43	1.26	1.63	1.43	1.26	1.63	Consequences of mistakes^a^
Pace of work dependent on speed of a machine or movement of a product	1.37	1.21	1.56	1.37	1.21	1.56	Factors of pace
No change of salary or income from January 2009	1.37	1.22	1.55	1.37	1.22	1.55	Changes in work situation^a^
No training paid for or provided	1.33	1.20	1.49	1.33	1.20	1.49	Training^a^
Monotonous tasks	1.32	1.19	1.46	1.29	1.17	1.43	Cognitive dimensions
Main job does not involve learning new things	1.32	1.19	1.47	1.28	1.15	1.42	Cognitive dimensions
More than 15 years in company or organization	1.32	1.18	1.47	1.32	1.18	1.47	Seniority^a^
Rarely consulted before targets for work are set	1.29	1.15	1.45	1.29	1.15	1.45	Decision latitude^a^
Not able to choose or change order of tasks	1.26	1.13	1.40	1.26	1.13	1.40	Control^a^

*OR* Odds ratio, *95% LCL* 95% lower confidence limit, *95% UCL* 95% upper confidence limit

^a^Not adjusted analysis per topic due to the availability of only a single indicator, bivariate analysis shown twice to improve the readability of the table

#### Analysis within topics

The results for logistic regressions analysis performed within each topic are also present in Table 1. In this analysis, seven variables from six groups were eliminated by the logistic regression procedure. The OR were lower for the following topics: ergonomic issues, job hazards, job security, work-life balance, working time and cognitive topics when compared to the bivariate analysis, respectively. The OR was higher for pace of work and remained the same for repetitive tasks when compared with their correspondent bivariate analysis. The variables analyzed showed mostly positive associations with lower limb pain, with the exception of two: (1) working with computers: PCs, network, mainframe, and (2) using internet/email for professional purposes.

#### Logistic model

The logistic regression procedure performed in one single equation of lower limb pain with all 39 variables eliminated 28. Thus, the remaining 11 variables defined our logistic model. The variables correspond to the following topics: ergonomic issues, work satisfaction, job hazards, job security, working time and repetitive tasks. The OR and CL of this model including the three demographic variables are presented in Table 2. The highest positive associations of lower limb pain were with: (1) tiring or painful positions, (2) carrying or moving heavy loads and (3) dissatisfaction with working conditions. Only one variable presented a negative OR with lower limb pain: working with computers.

**Table 2:** Associations between lower limb pain, working conditions and demographics from the fifth European Working Conditions Survey 2010 (27 EU countries)

Variable	Analysis model 20% sample			Analysis model 80% sample			Topics
	OR	99% LCL	99% UCL	OR	99% LCL	99% UCL	
Tiring or painful positions	1.74	1.50	2.00	2.02	1.88	2.17	Ergonomic issues
Carrying or moving heavy loads	1.61	1.37	1.88	1.48	1.37	1.60	Ergonomic issues
Standing	1.35	1.18	1.56	1.50	1.41	1.61	Ergonomic issues
Working with computers	0.71	0.60	0.84	0.74	0.68	0.80	Ergonomic issues
Dissatisfaction with working conditions	1.56	1.34	1.83	1.62	1.50	1.75	Work satisfaction
High temperatures	1.55	1.35	1.79	1.31	1.22	1.41	Job hazards
Breathing in vapors	1.40	1.22	1.62	1.28	1.19	1.38	Job hazards
Disagree: “I am well paid for the work I do”	1.39	1.22	1.58	1.36	1.28	1.45	Job security
Disagree: it would be easy for me to find a job of similar salary	1.31	1.15	1.50	1.11	1.04	1.19	Job security
Work in the evening more than five times a month	1.26	1.10	1.45	1.19	1.11	1.27	Working time
Short repetitive tasks of less than 10 min	1.25	1.11	1.42	1.18	1.11	1.25	Repetitive tasks
Gender (females vs males)	1.25	1.11	1.41	1.31	1.23	1.39	Demographics
No or only primary education	1.19	1.04	1.37	1.18	1.11	1.27	Demographics
Age (older vs younger)	1.87	1.64	2.13	1.81	1.70	1.93	Demographics

*OR* Odds ratio, *99% LCL* 99% lower confidence limit, *99% UCL* 99% upper confidence limit

### Model validation

The results of the model validation using the remaining 80% of the data are presented in Table 2. The strongest associations with lower limb pain in this case were with: (1) tiring and painful positions, (2) dissatisfaction with working conditions and (3) standing. Similar OR and CL were observed for both data samples among the variables. However, for the following variables: (a) tiring or painful positions, (b) carrying or moving heavy loads, (c) high temperatures, (d) breathing in vapors (e) standing and (f) it would be easy for me to find a job of similar salary (disagree), the 80% subsample OR values where outside the 20% subsample confidence limits and vice versa. Among the demographic variables, the highest associations were with age in both data sets.

Although the developed model (here tested with the full data set) was highly significant (Likelihood Ratio *χ* ^2^ = 3969.2; degrees of freedom, 14; *p* < 0.0001 the maximized *R* ^2^ (Nagelkerke 1991) only reached 0.173. Checking the model for each of the 27EU countries revealed that the model was highly significant but ranges of single ORs were large. For example, for tiring and painful position ORs lay between 1.34 and 2.72, for standing between 1.05 and 2.34, and for gender between 0.74 and 2.09.

### Impact of the model

#### Work-related cases and exposures

From the complete (*N* males = 17,466; *N* females = 17,906) EWCS 2010 data set, 31.7% male and 33.9% female respondents reported suffering from lower limb pain within the last 12 months. The percentage of lower limb pain cases attributable to working conditions when the respondents are exposed to different numbers of risk indicators (1 to ≥6) from the present model are presented in Table 3 for each gender along with the non-work-related lower limb pain cases. For this analysis only the model variables with positive associations with lower limb pain where considered, thus “working with computers” was excluded. In addition, the EWCS cross-national selection probability weighting was considered. The prevalence of work-related lower limb pain was 16.5% for males and 15.9% for females. Among the 5040 lower limb pain cases for males and 4072 for females 2759 of the male cases were judged to be work-related and 2215 of the female cases. The results showed that the absolute number of work-related lower limb pain cases is the highest with an exposure to more than six risk indicators for male respondents and four risk indicators for female respondents. In addition, most workers report to be exposed to one (males = 21%; females 26%) or two (males = 21%; females = 21%) risk indicators. Moreover, the relative risks for male and female respondents are reported in Table 3. Overall, the relative risks were higher for females than males.

**Table 3:** Total lower limb pain cases and work-related cases from the fifth European Working Conditions Survey 2010 (27 EU countries) when exposed to different number of risk indicators, for (a) males and (b) females

Number of risk factors present	*N*	Total leg pain cases	95% LCL	95% UCL	Work-related leg pain cases	Relative risk	95% LCL	95% UCL
(a) Male								
0	2240	305	0.12	0.15	0	1		
1	3448	585	0.16	0.18	115	1.25	1.10	1.42
2	3483	823	0.22	0.25	348	1.74	1.54	1.96
3	2633	858	0.30	0.34	500	2.39	2.13	2.69
4	2034	825	0.38	0.43	548	2.98	2.65	3.35
5	1351	651	0.46	0.51	467	3.54	3.14	3.98
≥6	1557	993	0.61	0.66	781	4.68	4.19	5.23
(b) Female								
0	2306	307	0.12	0.15	0	1		
1	3601	666	0.17	0.20	187	1.39	1.23	1.57
2	2861	724	0.24	0.27	343	1.90	1.68	2.15
3	2041	695	0.32	0.36	423	2.56	2.27	2.89
4	1493	681	0.43	0.48	482	3.43	3.04	3.86
5	794	425	0.50	0.57	319	4.02	3.56	4.55
≥6	847	574	0.65	0.71	461	5.09	4.54	5.70

Males: *N* = 16,746, frequency missing = 2629, Females: *N* = 13,942, frequency missing = 2055. Weighted according to the EWCS cross-national selection probability weighting

#### Lower limb pain and physical load

The variables related to physical loads (ergonomic issues topic) were used in a further frequency analysis. These variables are: (1) tiring or painful positions, (2) carrying and moving heavy loads, (3) standing, and (4) lifting or moving people. In addition, the EWCS cross-national selection probability weighting was considered. The prevalence of work-related lower limb pain due to physical loads was 10% for males and 10.5% for females. Thus, regarding physical loads, the number of work-related lower limb pain cases was 1926 males and 1661 females. Work-related lower limb pain cases increases when more than three physical risk indicators are present in males and two physical risk indicators in females (Table 4). In addition, the relative risks were higher for females than for male respondents.

**Table 4:** Total lower limb pain cases and work-related cases from the fifth European Working Conditions Survey 2010 (27 EU countries) according to the number of physical risk indicators present, for (a) males and (b) females

Number of physical risk factors present	*N*	Total leg pain cases	95% LCL	95% UCL	Work-related leg pain cases	Relative risk	95% LCL	95% UCL
(a) Male								
0	9564	1936	0.19	0.21	0	1		
1	5201	1613	0.30	0.32	560	1.53	1.45	1.62
2	2724	1256	0.44	0.48	705	2.28	2.15	2.41
≥3	1738	1013	0.56	0.61	661	2.88	2.72	3.04
(b) Female								
0	8042	1554	0.18	0.20	0	1		
1	4746	1512	0.31	0.33	595	1.65	1.55	1.75
2	2022	998	0.47	0.52	607	2.55	2.40	2.72
≥3	1067	665	0.59	0.65	459	3.23	3.02	3.44

Males: *N* = 19,228, frequency missing = 147. Females: *N* = 15,878, frequency missing = 120. Weighted according to the EWCS cross-national selection probability weighting

#### Work-related lower limb pain in the EU

In the 27 countries of the EU, 114,597,000 men and 95,145,000 women were employed during the study period according to Eurostat (2010). Based on this study, 30.1% of males and 29.2% of females suffered from lower limb pain; considering the EWCS cross-national selection probability weighting. Approximately half of the cases could be attributed to working conditions, for both genders. Thus, we estimate that in the 27 EU countries more than 18 million men and more than 15 million women suffered from work-related lower limb pain in 2010. Considering only the physical load, we estimate that more than 11 million men (10%) and more than 9 million women (10.5%) suffered from work-related lower limb pain due to physical loads at work in the 27 EU countries.

#### Associations with other health problems

Table 5 presents the percentage of respondents with other health problems according to the presence or absence of lower limb pain. The association of lower limb pain with other health-related variables showed the highest OR with muscular pains in shoulders, neck and/or upper limbs, and backache. Adjusted ORs for gender and age presented very similar values than those not adjusted (Table 5). Lower limb pain was also highly correlated with overall fatigue.

**Table 5:** Bivariate and adjusted associations of lower limb pain with other health problems

Variable	Leg pain (%)		OR	95% LCL	95% UCL	Adjusted age and gender		
	Yes	No				OR	95% LCL	95% UCL
Muscular pains in shoulders, neck and/or upper limbs	77.80	28.07	8.98	8.52	9.46	8.8	8.35	9.28
Backache	76.18	32.67	6.59	6.27	6.93	6.45	6.13	6.79
Respiratory difficulties	12.29	4.06	3.32	3.04	3.61	3.21	2.94	3.49
Overall fatigue	62.28	31.16	3.65	3.48	3.82	3.68	3.51	3.86
Injury	15.92	5.56	3.22	2.98	3.47	3.49	3.24	3.77
Cardiovascular diseases	12.07	3.83	3.45	3.16	3.76	3.00	2.74	3.28
Depression or anxiety	18.98	7.57	2.86	2.68	3.06	2.84	2.65	3.04
Skin problems	13.79	5.98	2.51	2.33	2.71	2.61	2.41	2.81
Insomnia or general sleep difficulties	31.87	14.83	2.69	2.55	2.83	2.59	2.46	2.73
Headaches, eyestrain	55.03	32.44	2.55	2.44	2.67	2.61	2.49	2.74
Hearing problems	10.89	4.64	2.51	2.31	2.73	2.34	2.15	2.55
Stomach ache	21.83	10.11	2.48	2.33	2.64	2.49	2.35	2.66

*N* = 35,372

#### Work expectations and absenteeism

For the male respondents with lower limb pain 56.25% believe they won’t be able to do the same job at 60 years of age, while 37.76% of the respondents without lower limb pain expected the same. The results were similar for the female respondents; 57.25% with lower limb pain believe they will not be able to do the same job at 60 years, while 39.92% without lower limb pain believe the same. The associations between lower limb pain and the expectation not to be able to do the same job at 60 years were 2.1 (95% CI 2.0–2.3) for males and 2.0 (95% CI 1.9–2.2) for females.

Among the respondents that reported lower limb pain, 23.6% males and 25.1% females were absent from work for more than 10 days in the last 12 months due to health problems as opposed to 12.9% of the males and 15.7% of the females without lower limb pain. However, 53.8% of the males and 51.8% of the females that reported lower limb pain did not miss any workdays due to health problems. In addition, 11.4% of the males and 5.9% of the females that reported lower limb pain were absent from work more than 10 days in the last year due to an accident at work as opposed to 4.1% of the males and 2.1% of the females without lower limb pain. 79.5% of the males and 89.3% of the females that reported lower limb pain did not miss any workdays due to an accident at work. This study estimates that over 146 million working days of male employees and 126 million working days of female employees were lost over the survey year due to lower limb pain in the EU.

## Discussion

By means of logistic regression analysis with backward elimination and using the EWCS 2010 data, this study identified ten indicators of working conditions that are combined with increased risk of lower limb pain. 30% of the EU27 workers experience lower limb pain which gives an estimate of 62 million (males: 34 million cases; females: 28 million cases) within a year. Based on this model 16% of the EU27 workers, 18 million male and 15 million female, suffered from lower limb pain linked to exposures at work. The more risk indicators reported, the higher the prevalence of lower limb pain. It increases from 17% at work situations without indicators of risk to 70% when six or more risk indicators are present. A substantial portion of the lower limb pain cases could be statistically linked to physical exposures at work such as standing, lifting, repetitive movements and awkward postures.

### Methodological considerations

The present study investigates risk indicators for lower limb pain from a wide range of topics related to working conditions using a large high quality representative random sample with a relatively high response rate (44%) drawn from the EU workforce. The EWCS has been carried out every five years since 1990 and its scope has increased substantially since its first launch. The large representative sample size and the numerous countries included in the EWCS offer a clear opportunity for this type of study and provide a good estimation of the impact of lower limb pain on the EU workforce, although the national languages translations may introduce some variance to the study. The EWCS data is cross-sectional and all limitations related to such studies apply, in particular the inability to determine casual relationships. It should also be noted that the data are based on self-reports: Exposures were not validated with independent measurements and health disorders were not diagnosed by clinical assessments. In addition, although the EWCS psychosocial questions have affinities with the well-established Karasek-Model and the Copenhagen Psychosocial Questionnaire (COPSOQ), they have not been validated through psychometric methods.

The EWCS questions use either Likert type scales or yes/no answers, thus it was possible to calculate odds ratios based on dichotomized variables. We chose to set the limit between exposed and not exposed in such a way that about one-fifth to one quarter was considered exposed and all the others were considered as controls. This has the advantage of focusing on the highest existing exposures at EU workplaces and of simplifying the analysis, because modelling of the dose–response relationship is avoided. However, it is acknowledged that this method biases toward conservative numerical risk evaluation, lacks evaluation of dose effects and provides no estimation of safe levels.

Finally, it should be noted that the present analysis focuses on the relationship between the reported work situation and lower limb pain. The analysis does not consider factors such as socioeconomic status, nationality, ethnicity, language, or occupational group. The present findings are relevant for the population of the total sample (EU27), it should be considered as a general model. Considerable deviations may occur when analyzing single countries or economic sectors, as noted by the high variability of the size of the ORs of single risk indicators among the EU27 countries. Last, with EWCS data we are not able to distinguish the specific region of the pain (hips, legs, knees, feet, etc.) as all are grouped in one category lower limb pain.

### Model validation

Due to the sheer number of potential predictors developed by logistic regressions, the resulting model may be biased. In an effort to better control this potential bias, a subsample of 20% was used for the development of the model and the remaining 80% was used for its validation. The magnitude of the calculated OR from both 20% and 80% subsamples were similar, but discrepancies were observed on six variables in that the corresponding OR values of the 80% subsample were outside the confidence limits obtained from the 20% subsample. These deviations show that the present model is not perfect; however, the included predictors were highly significant in the replication with the 80% subsample. To our knowledge, this is the first study that aims to develop a model of risk indicators for lower limb pain.

### Prevalence and risk indicators for lower limb pain

A high prevalence of lower limb pain was observed among the EWCS 2010 respondents and it strongly differed, depending on the number of risk indicators, rising from 17% when all ten risk indicators were negative to more than 70% when six or more were positive. Lower limb pain was associated with working-condition variables from the topics: ergonomic issues, job hazards and job security. Previous studies have found that poor ergonomic design, working in hazardous exposures and/or psychological factors may contribute to an increase of sickness absences, physical symptoms and/or musculoskeletal disorders (García-Herrero et al. 2012; Laaksonen et al. 2010; Yue et al. 2014). However, the evaluation of such factors with lower limb pain has received little attention.

The present study produced a model for lower limb pain that includes ten working- condition variables with positive associations and one (working with computers) with negative. It is reasonable to suppose that working with computers may be related to office work, where lower limb pain may be less prevalent than in other types of jobs. In the model, the topic “ergonomic issues” included the variables with the highest positive associations with lower limb pain. These correspond to work that requires physical loading. These findings corroborate a similar epidemiology study where exposures to tiring postures, carrying heavy loads and standing were also associated with lower limb symptoms in major occupational groups of the EWCS (Montano 2014). In addition, the present study revealed that when exposed to three physical risks indicators, lower limb pain is almost four times more probable in women and more than three times in men than without these exposures. Previous studies have shown an association between physical loading, such as tiring postures, standing and/or carrying heavy loads, with musculoskeletal symptoms in the ankle/foot, lower leg, knee and hips (Elsner et al. 1996; Messing et al. 2008; Riddle et al. 2003; Sandmark et al. 2000; Sulsky et al. 2012; Werner et al. 2010). These results support the view that ergonomic issues at work are a priority for the prevention of work-related lower limb health problems.

Work satisfaction and job insecurity, as presented in our model, have been previously associated with musculoskeletal symptoms and health issues. Low job satisfaction was related to symptoms in the low back and lower limbs in studies with teachers and manufacturing workers (Gell et al. 2011; Yue et al. 2014). Review papers have emphasized the strong associations between job insecurity with both mental health and physical symptoms (Kim and von dem Knesebeck 2015; Sverke et al. 2002). However, the specific association of job insecurity and lower limb pain has not been investigated before. Our findings suggest that satisfaction with the perceived working conditions and a lack of job security may also be important in the prevention of lower limb pain.

Many studies have shown an association between working conditions that involve exposures to hazardous chemicals, extreme temperatures, working at night and/or performing repetitive tasks with health problems (Ramin et al. 2015; Ross et al. 2016). However, very few studies have investigated the impact of these risks indicators on lower limb pain. A recent study found a significant association of lower limb pain with extreme temperatures and night shifts among workers of a processing plant (Barro et al. 2015). Performing repetitive tasks may increase the prevalence of MSDs such as upper extremity pain and low back pain (Andersen et al. 2007; Latza et al. 2002; Roelen et al. 2008; Tissot et al. 2005). However, there is little research that investigates the direct association of repetitive tasks with lower limb pain. One study found association of repetitive movements and extreme temperatures with lower limb pain in major occupational groups (Montano 2014). The present study suggests that repetitive tasks, job hazards and night shifts may be risk indicators for work related lower limb pain and should be considered in its prevention.

### Work-related lower limb pain

The findings of this study show that more than 50% of the lower limb pain cases could be attributed to work. Work-related lower limb pain cases increase when individuals are exposed multiple risk indicators. The corresponding risks ratio triples if the individual is exposed to more than six risk indicators compared to only one. However, the number of respondents exposed to more than three risk indicators in males and two risk indicators in females was relatively small, and jobs where workers are simultaneously exposed to all ten risk indicators are rare. Prevention should therefore focus on working situations where several risk indicators concur. The findings showed that the relative risks for males and females were similar. However, lower limb pain was slightly more often reported by females than males, which corroborates with Montano (2014) study.

### Lower limb pain, health problems and absenteeism

The high association of lower limb pain with upper limb symptoms and back pain is not surprising, as work with exposures to physical risk factors may affect diverse areas of the musculoskeletal system. A study found that both upper and lower limb symptoms are more common among craft workers, machine operators and workers in elementary occupations (Montano 2014). Another cross-sectional study revealed that end-of-line assembly workers have higher levels of complaints of pain in upper limbs, lower limbs and the back when compared to other workers (Chee and Rampal 2004). Improving the working conditions of lower limb pain sufferers may have a positive impact on other musculoskeletal symptoms. Although research studies focus more on the upper body, lower limb pain also needs attention. The present shows a substantial impact of such symptoms on absenteeism and the workers’ expectations as to their future ability to perform their job. Absenteeism is a relevant measure to evaluate employee health status in the job environment and is an indication of the economic and social costs of work-related MSDs (Bevan 2015; Laaksonen et al. 2010).

## Conclusion

This study is one of the few that have focused on lower limb pain and developed a model, based on working-condition indicators that include both physical and psychosocial aspects. The present model was validated and is capable of predicting the prevalence of lower limb pain in working populations, although it may contain a moderate level of conservative bias. The analysis estimated a prevalence of 30.1% for males and 29.2% of lower limb pain. Half of those cases are attributable to working conditions, where most cases are attributed to physical risks. More than 270 million days of absence from work can be explained by lower limb pain. Finally, the correlation of lower limb pain with other health problems may be explained by similar exposures at the same hazardous work places. Prevention strategies for work-related health problems should acknowledge the relevancy of lower limb pain and may focus on improving workplaces with multiple risks.
