# Correction to: Lower limb pain among workers: a cross-sectional analysis of the fifth European Working Conditions Survey

**DOI:** 10.1007/s00420-018-1313-8

**Published:** 2018-05-02

**Authors:** Maria-Gabriela Garcia, Margaret Graf, Thomas Läubli

**Affiliations:** 1 Sensory-Motor Systems Lab, Department of Health Sciences and Technology, ETH Zürich, Sonneggstrasse 3, ML G53.2, 8092 Zurich, Switzerland; 2 State Secretariat of Economic Affairs SECO, Bern, Switzerland; 3 Institute of Occupational and Social Medicine and Health Services Research, University of Tübingen, Tubingen, Germany

## Correction to: Int Arch Occup Environ Health (2017) 90: 575–585 10.1007/s00420-017-1220-4

The article “Lower limb pain among workers: a cross‑sectional analysis of the fifth European Working Conditions Survey”, written by Maria‑Gabriela Garcia, Margaret Graf, Thomas Läubli was originally published electronically on the publisher’s internet portal (currently SpringerLink) on 17 April, 2017 without open access.

With the author(s)’ decision to opt for Open Choice the copyright of the article changed on 25 April to © The Author(s) 2018 and the article is forthwith distributed under the terms of the Creative Commons Attribution 4.0 International License (http://creativecommons.org/licenses/by/4.0/), which permits use, duplication, adaptation, distribution and reproduction in any medium or format, as long as you give appropriate credit to the original author(s) and the source, provide a link to the Creative Commons license and indicate if changes were made.

The original article has been corrected.

